# Verbal Training Induces Enhanced Functional Connectivity in Japanese Healthy Elderly Population

**DOI:** 10.3389/fnhum.2022.786853

**Published:** 2022-03-03

**Authors:** Fan-Pei Gloria Yang, Tzu-Yu Liu, Chih-Hsuan Liu, Shumei Murakami, Toshiharu Nakai

**Affiliations:** ^1^Department of Foreign Languages and Literature, National Tsing Hua University, Hsinchu, Taiwan; ^2^Center for Cognition and Mind Sciences, National Tsing Hua University, Hsinchu, Taiwan; ^3^Department of Radiology, Graduate School of Dentistry, Osaka University, Suita, Japan; ^4^Institute of NeuroImaging and Informatics, Obu, Japan

**Keywords:** fMRI, aging, connectivity, plasticity, hippocampus, rsfMRI = resting state fMRI

## Abstract

This study employs fMRI to examine the neural substrates of response to cognitive training in healthy old adults. Twenty Japanese healthy elders participated in a 4-week program and practiced a verbal articulation task on a daily basis. Functional connectivity analysis revealed that in comparison to age- and education-matched controls, elders who received the cognitive training demonstrated increased connectivity in the frontotemporal regions related with language and memory functions and showed significant correlations between the behavioral change in a linguistic task and connectivity in regions for goal-oriented persistence and lexical processing. The increased hippocampal connectivity was consistent with previous research showing efficacious memory improvement and change in hippocampal functioning. Moreover, the increased intra-network connectivity following cognitive training suggested an improved neural differentiation, in contrast to the inter-network activation pattern typical in the aging brain. This research not only validates the relationship of functional change in the frontal and temporal lobes to age-associated cognitive decline but also shows promise in turning neural change toward the right direction by cognitive training.

## Introduction

With the increasing aging population around the globe and the lack in effective treatment in dementia, measures to intervene cognitive decline are urgently needed. Cognitive training is one of the strategies that has shown some promise in the retention of cognitive functions for healthy elders and elders at risk for mild cognitive impairments (MCI) (Ball et al., 2002; Belleville, 2008; Valenzuela and Sachdev, 2009; Mowszowski et al., 2010; Rosen et al., 2011). Additionally, studies have suggested that enriching mental activities could moderate the deterioration process as healthy elders participating in social and cognitive activities were less likely to develop MCI and eventually converted to dementia (Wilson et al., 2002; Verghese et al., 2003, 2006; Prince et al., 2013). A recent review on non-pharmaceutical interventions based on 13 reviews or meta-analyses published from 2010 to 2019 reported that cognitive interventions focusing on memory or language skills, executive functions, social interactions, etc. were effective for maintaining or improving cognitive functions in older adults regardless of their cognitive status (Sanjuan et al., 2020). This raises the question of which type of language-based training is effective on older adults who are cognitively normal.

The transmission deficit hypothesis (TDH) has pointed out that the change in linguistic competence is asymmetric in aging, with comprehension relatively preserved and production strongly affected (Burke and Shafto, 2004). Behavioral evidence has shown that word retrieval problem in healthy aging is due to a failure in the complete retrieval of the phonology of the target word, instead of generalized slowing (Shafto et al., 2007). In another study using event-related potential (ERP) and visual evoked potential (VEP), healthy young and older adults performed an implicit picture-naming task while making a segmental or syllabic decision in a Go/No-go paradigm. The older adults showed longer latencies in both behavioral judgment and ERP amplitudes in response to phonological stimuli. In contrast, there was no latency difference between the young and old groups regarding the VEP stimuli. The authors concluded that age-associated delay was only specific to the phonological system and proposed that the practice of phonological skills might improve general linguistic abilities in older adults (Neumann et al., 2009). This study hypothesizes that a linguistic task focusing on articulatory skills will improve general linguistic abilities for two reasons. First, appropriate articulation calls for awareness of linguistic constituents, which are fundamentals for proper word and sentence production. Second, articulatory practice will activate the cortices and connectivity related to phonological retrieval, which has been reported to be the cause of production difficulty and word retrieval problem commonly seen in older adults (Shafto et al., 2007).

The temporal regions, particularly the medial portions, including the hippocampus, were most commonly impacted by neurodegeneration, as studies of MCI and early Alzheimer’s disease (AD) have consistently revealed hippocampus atrophy (Braak and Braak, 1996; Kordower et al., 2001; Scheff et al., 2006). The hippocampus is related to conscious memory recollection, and the hippocampal lesions are associated with memory deficits in MCI and AD (Cohen and Squire, 1981; Tulving, 2002). Improvements in memory and change in hippocampal functioning could suggest alterations in the disease progression of MCI and AD. Therefore, interventions leading to the functional change in the hippocampus might be considered as an effective strategy for retention of cognitive reserve in successful aging. This study predicts the change in the functional connectivity in the hippocampus and sensory-motor regions after articulation training, as the training task taps into the cortices associated with movement and verbal memory. The frontal lobe is also anticipated to show improved connectivity as most previous studies have revealed frontal involvement when cognitive interventions are effective (Sanjuan et al., 2020).

Cognitive neuroscience of aging has addressed the relationship between cognitive function performance with global and local functional activations in the brain. The increased bilateral, mostly prefrontal involvement has been consistently reported in healthy old adults, while other regions either showed decreased or increased activations relative to those in young adults, as discussed in a review (Park and Reuter-Lorenz, 2009). The localized activation difference from the young adults was often interpreted as compensation or degeneration, with conflicting results by different experimental manipulations. Such inconsistency used to be attributed to the paradigm or task demand, with a weak foundation for the argument reasoning. This problem suggested that the investigation of isolated regions in the brain may not suffice to understand the neural change associated with age-induced decline. Therefore, there has been a support for the examination of network connectivity for interacting regions to elucidate the neural bases underlying altered cognitive functions in aging (Antonenko and Floel, 2014).

Functional connectivity (FC) between brain regions reveals the degree of synchronization or temporal correlations of activities in them. It reflects the quality of information transfer among brain regions, which could be achieved by direct white matter pathways or indirect connections through multiple regions (Fox and Raichle, 2007). Previous research has related hyper- and hypo-connectivity in older adults compared with young adults to decreased cognitive functions (Antonenko and Floel, 2014). Various techniques, such as electroencephalography, magnetoencephalography, and functional magnetic resonance imaging (fMRI), have been employed to study functional connectivity (Buldu et al., 2011; Ferreira and Busatto, 2013; Freitas et al., 2013). FC has been analyzed by means of correlational (Fox and Raichle, 2007), non-linear (Buldu et al., 2011), and graph-theoretical approaches (Meunier et al., 2009). This study focuses on the resting-state fMRI connectivity, similar to those in the review of FC approaches (Ferreira and Busatto, 2013), using the inter-regional correlational approach.

## Materials and Methods

### Participants

Twenty healthy elders (mean age = 69.7, *SD* = 4.2, 8 women) participated in the cognitive training group, and 20 age-matched controls (mean age = 70.3, *SD* = 3.6, 10 women) enrolled in this study. As shown in Table 1, there was no significant difference between the training and control groups in the education (training group mean = 12.8, *SD* = 2.5; control group mean = 11.9, *SD* = 2.1), WAIS III Vocabulary Scaled Score (training group mean = 11.8, *SD* = 2.7; control group mean = 12.5, *SD* = 2.5), Mini-Mental State Examination (MMSE) (training group mean = 29.0, *SD* = 1.5; control group mean = 29.4, *SD* = 0.8), geriatric depression scale (GDS) (training group mean = 2.1, *SD* = 2.3; control group mean = 0.9, *SD* = 1.2), and H.N. Handedness Test (training group mean = 97.0, *SD* = 9.5; control group mean = 99.5, *SD* = 2.4).

**TABLE 1:** Baseline scores for the clinical and demographic characteristics of the control and cognitive training groups.

	Training (*n* = 20)	Control (*n* = 20)		
**Characteristics^a^**			***F (df)***	***p*-value**
Age, years	69.7 (4.2)	70.3 (3.6)	0.5107 (1, 39)	0.479092
Education, years	12.8 (2.5)	11.9 (2.1)	0.3635 (1, 39)	0.550059
WAISS lll Vocabulary (Raw score)	31.5 (10.1)	33.4 (10.2)	0.9614 (1, 39)	0.3328781
WAISS lll Vocabulary (Scaled score)	11.8 (2.7)	12.5 (2.5)	0.7404 (1, 39)	0.3947919
MMSE (total raw, /30)	29.0 (1.5)	29.4 (0.8)	0.0068 (1, 39)	0.934701
GDS (total raw, /30)	2.1 (2.3)	0.9 (1.2)	0.0071 (1, 39)	0.9332796
H.N. Handedness Test	97.0 (9.5)	99.5 (2.4)	1.3315 (1, 39)	0.255561
**Characteristics^b^**			**x^2^**	***p*-value**
Gender, female (% of total subjects)	8 (20)	10 (25)	0.4	0.5270893

*^a^Data reported as mean (SD); ^b^Data reported as number (%). MMSE, Mini-Mental State Examination. GDS, Geriatric Depression Scale. The whole study lasted 5 weeks. During the training period (Day 2–Day 27), participants were instructed to complete the verbal articulation task by reading 40 sentences (10 for each condition) 10 times as fast and accurately as possible. The training time lasted 20–25 min/day, with a 1-day break after the 3-day training.*

### Procedure

**FIGURE 1:**
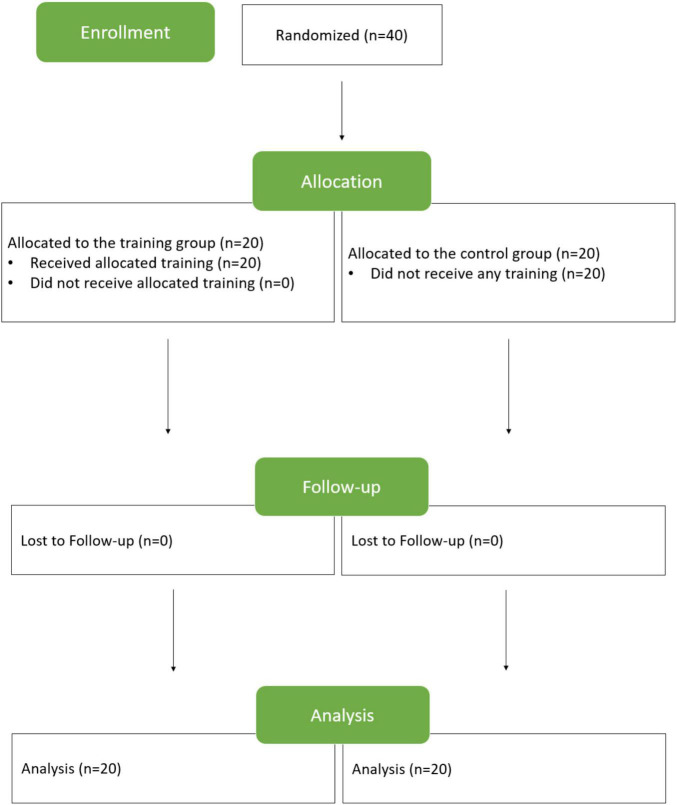
Study procedure.

The study procedure is illustrated in Figure 1. After healthy elders were recruited, they were randomly assigned to the training group or control group. The training group was offered explanations of behavioral measurements and scans and the training program, including the training stimuli, program duration, and practice frequency. All the 20 elders agreed to complete the program and were allocated to individual training sessions. The participants assigned to the control group were provided with explanations of behavioral measurements and scans. All of them agreed to participate in the study. Within the 28-day period of the study, research assistants made phone calls to inquire about their willingness to stay in the study and keep elders motivated by encouraging them. No elders dropped out of the study. Behavioral and scan data of all subjects collected on days 1 and 28 were analyzed.

At baseline (Day 1), all participants underwent scanning, including MPRAGE (6 min), resting-state fMRI (7 min), and DTI (8 min). Later, they completed a standardized battery of neuropsychological tests including the verbal fluency test (5 min), Stroop task (10 min), Bochumer Matrices Test (BOMAT) (15 min), WAIS-III digit span (backward only, 5 min), WAIS-III digit symbol (5 min), MMSE (10 min), GDS (3 min), H.N. Handedness (3 min), and a sentence reading task (10 min). The sentences used in the sentence reading task were categorized by sensicality and difficulty into four types: easy non-sensical sentences, difficult non-sensical sentences, easy meaningful sentences, and difficult meaningful sentences. Participants were asked to read 10 items in each type, 40 items in total. Durations of reading four types of sentences were documented for all subjects.

**FIGURE 2:**
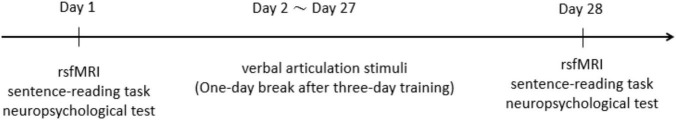
Timing diagram of the scans and stimulus delivery.

During the period of training, from Days 2 to 27, participants were instructed to complete the verbal articulation of 40 sentences (10 for each condition). They were required to read aloud each sentence 10 times as fast and accurately as possible, with the reading being recorded. The training time lasted 20–25 min/day, with a 1-day break after the 3-day training. Furthermore, participants were checked whether they performed the task properly by phone every 4 days. On Day 28, all participants underwent the scans, neuropsychological battery testing, and the sentence-reading task. The timing arrangement for behavioral tests, sentence reading tasks, rsfMRI, and verbal articulation training is represented in Figure 2.

### Verbal Articulation Training

There is evidence showing that the articulation rate and articulation stability are affected in aging (Tremblay et al., 2019). Particularly, aging is associated with an increase in the duration and duration variability of speech utterances in a number of tasks, such as syllable and non-word reading (Tremblay and Deschamps, 2016; Tremblay et al., 2017, 2018), and non-word repetition (Sadagopan and Smith, 2013). This study employs verbal articulation training as the intervention for two reasons. First, the training is expected to improve the articulation in elders, which they have already experienced difficulties in and might feel frustrated within daily life. Second, the practice of articulation skills is anticipated to influence the performance of other linguistic tasks, as previous studies proposed that failure in phonological retrieval led to other linguistic difficulties, such as word retrieval difficulties and delayed response (Burke and Shafto, 2004; Shafto et al., 2007; Shafto and Tyler, 2014), and suggested that elders might benefit from the training of phonological skills (Neumann et al., 2009).

The psychometric properties of the verbal articulation training included comprehension, language, memory, or articulation functions. The stimuli of the verbal articulation training contained four sets of sentences. The first set was difficult-to-articulate sentences with real words. The second set was an easy-to-articulate sentence with real words. The third set was a difficult-to-articulate sentence with pseudowords. The final set was an easy-to-articulate sentence with pseudowords. The definition of difficult or easy to articulate was determined by the consonants which were late or early acquired by Japanese children. To exclude any confounding factors, the syntactic structure, morae number, word familiarity, and word imageability were matched across the four conditions. All sentences were presented in the order of Subject-Adjective-Object-Verb. Words, such as wa, no, and wo appearing in sentences, were case markers for syntactic features. A typical example for the first set of training stimuli was シホは　私費の 施設を 保守する “Shiho protects a private facility.” An example for the second set was マキは 棚の 刀を 磨く “Maki polishes a sword on the shelf.” In the third and final sets with pseudowords, the sentences also followed an order of Subject-Adjective-Object-Verb with easy or difficult-to-articulate consonants. Table 2 illustrates the weekly training schedule with training and break days.

**TABLE 2:** The training schedule.

	Wed/Sat	Thu/Sun	Fri/Mon	Sat/Tue	Sun/Wed	Mon/Thu	Tue/Fri
Week 1	fMRI-1 Behav-1	Train-1	Train-2	Train-3 (call)		Train-4	Train-5
Week 2	Train-6 (call)		Train-7	Train-8	Train-9 (call)		Train-10
Week 3	Train-11	Train-12 (call)		Train-13	Train-14	Train-15 (call)	
Week 4	Train-16	Train-17	Train-18 (call)		Train-19	Train-20	Train-21 (call)
Week 5	fMRI-2 Behav-2						

### Image Acquisition

Images were acquired in a 3 Tesla MR scanner (TIM Trio, Siemens, Erlangen, Germany) with a 12-channel head coil in National Center for Geriatrics and Gerontology. A gradient-echo echo planar imaging (EPI) sequence was used for functional images to measure blood-oxygen level-dependent (BOLD) contrast with the following parameters: repetition time (TR) = 3,000 ms, echo time (TE) = 30 ms, flip angle = 90°, field of view (FOV) = 192 mm, voxel size = 3 × 3 mm, slice thickness = 3 mm, and 39 axial slices with 0.75 mm gaps. A T2-weighted image (TR = 5,920 ms, TE = 95 ms, flip angle = 150°, FOV = 192 mm, voxel size = 0.8 × 0.8 mm, slice thickness = 3 mm, and 39 axial slices with 0.75-mm gaps). For anatomical references, a high-resolution T1-weighted 3D MPRAGE scan covering the whole brain (TR = 2,500 ms, TE = 2.63 ms, flip angle = 7°, FOV = 256 mm, and isotropic voxels 1 × 1 × 1 mm) was obtained for all participants.

### Resting-State Functional Magnetic Resonance Imaging Processing

We used the CONN functional connectivity toolbox (version 16.a),^1^ in conjunction with SPM12 (Wellcome Department of Cognitive Neurology, London, United Kingdom),^2^ to perform all preprocessing steps (using default preprocessing pipeline of CONN) and subsequent statistical analyses. In this preprocessing pipeline, raw functional images were slice-time corrected, realigned (motion-corrected), unwrapped, and coregistered to the MPRAGE image of each subject in accordance with standard algorithms.

Denoising pipeline of CONN employed linear regression to estimate and remove potential confounding factors for each voxel for each subject and functional run/session and then used temporal band-pass filtering to remove temporal frequencies below 0.008 Hz or above 0.09 Hz to focus on slow frequency fluctuations. Potential confounding factors included noise components from cerebral white matter and cerebrospinal areas (Behzadi et al., 2007), estimated subject-motion parameters (Friston et al., 1996), identified outlier scans or scrubbing (Power et al., 2014), constant and first-order linear session effects, and constant task effects (Whitfield-Gabrieli and Nieto-Castanon, 2012). To minimize the BOLD variability caused by head motion, 12 potential noise components were defined from the estimated subject-motion parameters, with 3 translation parameters, 3 rotation parameters, and their associated first-order derivatives.

Images were then normalized to Montreal Neurological Institute coordinate space, spatially smoothed (8-mm full-width at half maximum), and resliced to 2 × 2 × 2 mm voxels. The regions of interest (ROIs) in this study were derived from a freely available ROI atlas defined by correlated activation patterns.^3^ All ROIs provided by the atlas were used for analysis.

### Functional Connectivity Measures and Statistical Analysis

For the calculation of FC, we used the ROI-to-ROI measure, which characterizes the connectivity between all pairs of ROIs among a predefined set of regions. The level of connectivity is represented by an ROI-to-ROI (PRC) matrix, in which each element is defined as the Fisher-transformed bivariate correlation coefficient between a pair of ROI BOLD time series:


(1) $$r\left(i,j\right)=\frac{\int R_{i}\left(t\right)R_{j}\left(t\right)dt}{{\left(\int R_{i}^{2}\left(t\right)dt\int R_{j}^{2}\left(t\right)dt\right)}^{1/2}}$$

(2) $$Z\left(i,j\right)=\tanh^{-1}\left(r\left(i,j\right)\right)$$

where *r* is a matrix of correlation coefficients, *R* is the BOLD time series within each ROI, and *Z* is the RRC matrix of Fisher-transformed correlation coefficients.

The reading time difference of the sentence-reading task of each subject was also added as 2nd level covariates. This yielded the brain maps demonstrating the correlation between the reading time and the post-functional > pre-functional connectivity. All group-level results were corrected for multiple comparisons (false discovery rate, FDR) (*p* < 0.05) within the CONN toolbox.

In studying the effect of training on the performance of the sentence-reading task, we conducted a three-way ANOVA test on reading speed of the verbal training group. The effects and interactions of three factors were examined, namely, training, sensicality of sentences, and difficulty of sentences. Table 3 shows the analysis of speed (duration) in reading sentences and the effects of training. In comparing the change of neuropsychological battery after 28 days in the training and control groups, we simply subtracted the 2nd measurement from the 1st measurement of these tests in two groups, respectively, and performed an *F* test on the subtraction difference between the two measurements.

**TABLE 3:** ANOVA of speed in reading sentences.

	Df	Sum sq	Mean sq	*F*-value	*p*-value
Training^a^	1	571,451,416	571,451,416	2633.989	<0.001***
Sensicality^b^	1	62,990,103	62,990,103	290.340	<0.001***
Difficulty^c^	1	1,231,336	1,231,336	5.6756	0.022*
Training: Sensicality	1	111,891,968	111,891,968	515.7434	<0.001***
Training: Difficulty	1	23,562	23,562	0.1086	0.741
Sensicality: Difficulty	1	6,770	6,770	0.0312	0.860
Training: Sensicality: Difficulty	1	1,478,534	1,478,534	6.8150	0.009**

*^a^Pretraining and post-training.*

*^b^Non-sensical and meaningful sentences.*

*^c^Easy and difficult sentences.*

*p < 0.05; **p < 0.01; ***p < 0.005.

## Results

### Behavioral Measures

The treatment effect was investigated with the sentence-reading task, neuropsychological battery, and questionnaire data by the *F-*test. As shown in Table 3, the ANOVA of reading non-sensical and meaning sentences revealed significant main effects of training (*p* < 0.001), sensicality (*p* < 0.001), and difficulty (*p* = 0.022) and interactions of training × sensicality (*p* < 0.001) and training × sensicality × difficulty (*p* = 0.009). The differences of the neuropsychological battery in the baseline and follow-up between the control and training groups did not reach significance between groups (cf. Supplementary Table 1).

### Post-functional Pre-functional Connectivity Change

**FIGURE 3:**
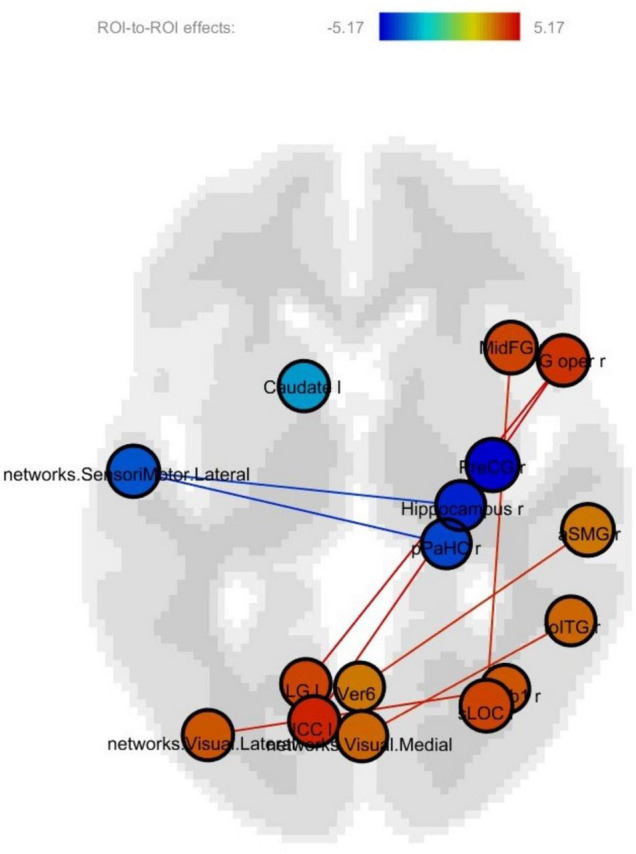
Post-training > pretraining connectivity.

The regions showing significant connectivity (*p* < 0.05, uncorrected) are represented in Figure 3 and Table 4. The color in the node circles represents the connectivity intensity, and the size of the node suggests the degree of hubness of the node, with the bigger size being the hub with a larger number of connections to other nodes. The edges are the lines connecting nodes, which represent the connectivity among ROIs. As shown in Figure 3, all the ROIs demonstrated a similar level of intensity. The ROIs with increased connectivity were mostly right-lateralized within the fronto-temporal network, e.g., the middle frontal gyrus, the inferior frontal gyrus opercularis, the hippocampus, parahippocampal gyrus, and the temporo-occipital part of the inferior temporal gyrus. These ROIs belong to the language and memory networks. The language network ROIs included the right inferior frontal gyrus opercularis and inferior temporal gyrus. The memory network ROIs consisted of the right hippocampus, right parahippocampal gyrus, and temporoparietal cortex. The temporal ROIs also exhibited connectivity with the left precentral gyrus, visual cortices, and the left inferior cingulate gyrus.

**TABLE 4:** Post-training > pretraining connectivity statistics.

Analysis Unit	*F*-value (df)	*p*-unc	*p*-FDR
Hippocampus r	3.20 (4.16)	**0.0413**	0.4503
Cereb1 r	6.03 (4.16)	**0.0037**	0.1656
MidFG r	2.1 (4.16)	0.1276	0.6345
IFG oper r	1.47 (4.16)	0.2561	0.6718
Caudate l	2.15 (4.16)	0.1218	0.6345
PreCG r	1.03 (4.16)	0.4198	0.7462
Networks.SensoriMotor.Lateral l	2.08 (4.16)	0.1317	0.6345
aSMG l	1.51 (4.16)	0.2467	0.6718
pPaHC r	3.18 (4.16)	**0.0421**	0.4503
toITG r	3.63 (4.16)	**0.0275**	0.4503
LG l	3.77 (4.16)	**0.0242**	0.4503
Ver 6	1.35 (4.16)	0.2939	0.6718
sLOC r	1.91 (4.16)	0.1587	0.6345
ICC l	4.57 (4.16)	**0.0119**	0.3181
Networks.Visual.Medial r	7.09 (4.16)	**0.0018**	0.1656
Networks.Visual.Lateral l	1.32 (4.16)	0.3066	0.6718

*Hippocampus r, the right hippocampus; Cereb1 r, the right cerebellum 1; MidFG r, the right middle frontal gyrus; IFG oper r, the right inferior frontal gyrus opercularis; Caudate l, the left caudate; networks.SensoriMotor.Lateral l, the left lateral motor cortex; PreCG r, the right precentral gyrus; aSMG l, the left anterior supramarginal gyrus; pPaHC r, the right parahippocampal gyrus; toITG r, the right temporo-occipital part of inferior temporal gyrus; LG l, the left lingual gyrus; Ver 6, Vermis 6; sLOC r, the right superior lateral occipital cortex; ICC l, the left inferior cingulate cortex; networks.Visual.Medial r, the right medial visual cortex; networks.Visual.Lateral l, the left lateral visual cortex. The bold values indicate p < 0.05.*

### Correlation Between Functional Connectivity and Reading Speed of the Sentence Reading Task

**FIGURE 4:**
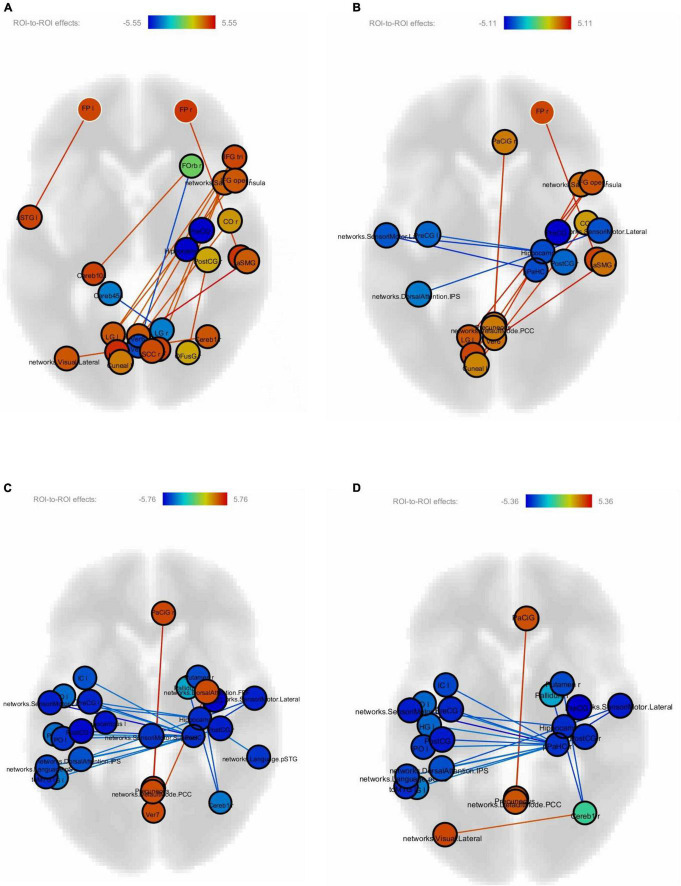
**(A)** Correlations between connectivity difference and reading speed of difficult non-sensical sentences. **(B)** Correlations between connectivity difference and reading speed of easy non-sensical sentences. **(C)** Correlations between connectivity difference and reading speed of difficult meaningful sentences. **(D)** Correlations between connectivity difference and reading speed of easy meaningful sentences.

Figures 4A–D show correlations between connectivity intensity and change in reading speed of the sentences without FDR correction. In this analysis, the behavior-function correlation was calculated by using the change of reading speed as a covariate in the computation of the regional intensity of each ROI and connectivity between ROIs. The nodes in color were the ROIs whose intensities correlated with the behavioral change, and the edges were the ROI-to-ROI connectivity showing correlations with the behavioral change. The frontal pole-planum temporal connectivity significantly correlated with the post-training > pre-training reading speed difference of the difficult non-sensical and easy non-sensical sentences (*p* < 0.05, FDR-corrected). There was an additional frontal pole-anterior superior temporal gyrus connectivity significantly correlated with the difficult non-sensical sentences (*p* < 0.05, FDR-corrected). There were no significant correlations after FDR correction between FC and the reading speed difference of easy and difficult meaningful sentences.

## Discussion

Although the reading speed of the sentence reading task has been shown to be affected by training, the scores of neuropsychological battery before and after training in the cognitive training group did not reach statistical significance. Neither did the pre- and post-change between the control and training groups reveal significant differences (Supplementary Table 1). A potential reason could be the lack of intensity in a simple task in this study. According to reviews and meta-analysis of interventions that have proven to be more effective in improving general functioning, the intervention programs have the following characteristics: (1) a minimum of 10 weeks of treatment with two sessions per week, (2) session length of 60–90 min, (3) interventions for healthy aging in a group format, (4) study follow-ups included, (5) several cognitive skills worked on at the same time, (6) inclusion of other components related to quality of life, (7) employment of personal, internal, or external strategies, and (8) inclusion of measurements of daily functioning (Sanjuan et al., 2020). The articulation task is a relatively simple task that does not include other components related to the quality of life, such as decreasing depression level and improvement of sleep quality, as suggested in the review research. An articulation task is not a challenging task that requires the assistance of internal or external strategies. Additionally, the training program in this research was in individual format and lacked group dynamics that could boost motivations and confidence to apply the skills to daily life functioning. Consequently, the linguistic improvement resulting from articulation training could not be translated to other behavior measured by the neuropsychological battery.

In contrast, the fMRI connectivity analysis revealed prominently increased connectivity in the ROIs within the language and memory networks in the frontal and temporal lobes after training. The temporal ROIs also exhibited connectivity with the left precentral gyrus, visual cortices, and the left inferior cingulate gyrus. The pathological change in the medial temporal lobe (MTL) has been found to be strongly associated with neurodegeneration in elders (Braak and Braak, 1996; Kordower et al., 2001; Scheff et al., 2006). Research has also reported an association of higher life-span cognitive activity with a reduced rate of hippocampal atrophy in elders at risk for dementia (Valenzuela et al., 2008). Studies that employed active training for healthy elders and effectively slowed down the progression to MCI have shown that training was most efficacious in improving memory (Verghese et al., 2003, 2006; Norrie et al., 2011; Rosen et al., 2011; Prince et al., 2013; Diamond et al., 2015). The enhanced hippocampal connectivity in this study is in accordance with previous research that revealed increased hippocampal activations in elders with MCI after cognitive training (Rosen et al., 2011).

The increased connections of the hippocampus to the cerebellum also suggested enhanced communications to achieve cognitive functions, as previous research has demonstrated a functional link between the cerebellum and hippocampus in humans to facilitate the cognitive aspects of navigation (Igloi et al., 2015). In a previous study, the task-based analyses using 787 subjects from the human connectome project showed that the cerebellar cortex was engaged in language, working memory, movement, social, and emotional task processing (Guell et al., 2018a). In this research, the cerebellum lobule showed increased connectivity with the temporal lobe, which is consistent with the observation that the non-motor processing lobule in the cerebellum supports a cognitive function that is left-lateralized, i.e., language (Guell et al., 2018b; Schmahmann et al., 2019).

Notably, in this study, there was right-lateralized increased connectivity within the frontotemporal network (Figure 3). This suggested that the effectiveness of the training was manifested in enhanced communications among the frontotemporal language regions (i.e., the inferior frontal gyrus opercularis and inferior temporal gyrus) and temporal memory regions (i.e., the hippocampus and parahippocampal gyrus) in the right hemisphere. Prior fMRI studies on healthy aging have generally reported spread instead of localized functional activations in elders relative to young controls (Salami et al., 2012; Spreng and Schacter, 2012). Inter-network connectivity or activations seem to suggest detrimental effects caused by age-related inefficiency, which is consistent with neural dedifferentiation in the aging brain (Park and Reuter-Lorenz, 2009; Antonenko and Floel, 2014; Geerligs et al., 2014). The increased intra-network connectivity following cognitive training in this research could serve as evidence for improved neural differentiation. This also provides a solid foundation for the rationale of turning neural change toward the right direction by cognitive training.

The correlation between FC in the frontal pole and improved performance of the sentence reading task suggested the important role of frontal involvement in driving the task success as a result of articulation training. Recent research has shown that the structural properties of the frontal pole cortex contain information that can differentiate participants with high goal-directed persistence from those with low persistence, regardless of task domains, such as cognitive, language, and motor learning (Hosoda et al., 2020). They also found that participants with high persistence exhibited experience-dependent neuroplastic changes in the frontal pole after completing language and motor learning tasks. The increased connectivity between the frontal pole and planum temporale, which is a region typically involved in auditory processing (Nakada et al., 2001) and lexical processing (Bookheimer, 2002), could also represent the post-training neural plasticity in the fontal pole to complete the task involving auditory and lexical processes. Additionally, the anterior superior temporal gyrus has been reported to be related to sentence processing (Mellem et al., 2016). The connectivity between the anterior superior temporal gyrus and the frontal pole might suggest the training-induced persistence in reading difficult non-sensical sentences.

Regarding maintenance of training effects, previous research has reported that sustained benefits of cognitive interventions were verified in healthy elders for periods of 2 months (Chiu et al., 2017) to 5 years (Kelly et al., 2014) after treatment. The sustainability of the effects also relied on subsequent practices of the training stimuli, similar to any kind of skills. The study follow-ups are crucial for not only maintenance of the effects but also translation of the skills to general functioning (Sanjuan et al., 2020). This study did not follow up with the participants after the study was completed. Our future study will take into consideration long-term follow-ups and measurements of sustained treatment effects.

## Conclusion

This study utilizes a simple verbal articulation task for cognitive training in Japanese healthy adults. In comparison to age- and education-matched controls, elders who received the articulation training demonstrated significantly increased connectivity in the right frontotemporal regions, with extended connectivity from temporal regions to cerebellum and visual cortices. The increased hippocampal connectivity was consistent with previous research showing efficacy in intervening cognitive decline and change in hippocampal functioning. Moreover, the increased intra-network connectivity following cognitive training suggested an improved neural differentiation, in contrast to the dedifferentiation pattern in the aging brain. Although the training was simply relative to programs used in other cognitive training studies, the fMRI connectivity analysis showed patterns suggesting a promising functional change in the frontal and temporal regions that are associated with goal-oriented persistence, as well as language and memory functions.

## Data Availability Statement

The original contributions presented in the study are included in the article/Supplementary Material, further inquiries can be directed to the corresponding author/s.

## Ethics Statement

The studies involving human participants were reviewed and approved by IRB of National Center for Geriatrics and Gerontology. The patients/participants provided their written informed consent to participate in this study.

## Author Contributions

F-PY analyzed the data and wrote the manuscript. T-YL and C-HL assisted in the preparation of tables and figures and the organization of references. TN helped in designing the task and organized data collection. SM provided opinions on the results of verbal training from the perspective of oral functions and worked with TN to supervise the collaborative research and review the manuscript draft. All authors contributed to the article and approved the submitted version.

## Conflict of Interest

The authors declare that the research was conducted in the absence of any commercial or financial relationships that could be construed as a potential conflict of interest.

## Publisher’s Note

All claims expressed in this article are solely those of the authors and do not necessarily represent those of their affiliated organizations, or those of the publisher, the editors and the reviewers. Any product that may be evaluated in this article, or claim that may be made by its manufacturer, is not guaranteed or endorsed by the publisher.

## Footnotes

1
https://www.nitrc.org/projects/conn

2
http://www.fil.ion.ucl.ac.uk/spm

3
http://findlab.stanford.edu/functional_ROIs.html
