# Supplementary material for: Verbal Training Induces Enhanced Functional Connectivity in Japanese Healthy Elderly Population
Source: Front Hum Neurosci. 2022 Mar 3;16:786853. doi: 10.3389/fnhum.2022.786853 (PMC8930077; doi:10.3389/fnhum.2022.786853)
Supplement: Supplementary file 1 [file Table_1.docx]

| Supplementary Table 1 | | | | | | | | | | | |  |  |  |  |  |  |  |  |
| --- | --- | --- | --- | --- | --- | --- | --- | --- | --- | --- | --- | --- | --- | --- | --- | --- | --- | --- | --- |
| Baseline and Follow-up neuropsychological and questionnaire data of the control and cognitive training groups | | | | | | | | | | | |  |  |  |  |  |  |  |  |
|  | Training (n=20) | | | Control (n=20) | | | | Overall | | | | |  |  |  |  |  |  |  |
|  | Baseline | Follow-up | Mean diff | Baseline | | Follow-up | Mean diff | *F (df)* | *p*-value | Cohen's *d** | | |  |  |  |  |  |  |  |
| RAVEN SPM (total raw, /36) | 22.2 (4.9) | 24.0 (5.7) | 1.8 (2.8) | 23.5 (4.8) | | 25.2 (4.8) | 1.7 (3.4) | 0.43 (1, 39) | 0.51583898 | 0.228 | | |  |  |  |  |  |  |  |
| Digit Symbol (total scaled, /19) | 12.7 (2.5) | 13.9 (2.2) | 1.2 (1.6) | 13.5 (2.8) | | 14.5 (2.7) | 1.0 (1.0) | 0.32 (1, 39) | 0.574848096 | 0.244 | | |  |  |  |  |  |  |  |
| Digit Span: forward (total raw, /16) | 8.6 (1.9) | 8.2 (1.7) | -0.4 (1.7) | 8.5 (2.2) | | 8.4 (2.1) | -0.1 (1.6) | 0.35 (1, 39) | 0.557527118 | 0.105 | | |  |  |  |  |  |  |  |
| Digit Span: backward (total raw, /14) | 5.0 (1.5) | 5.7 (1.8) | 0.7 (1.6) | 5.5 (1.7) | | 5.0 (2.4) | -0.5 (1.6) | 0.23 (1, 39) | 0.634202437 | **0.33** | | |  |  |  |  |  |  |  |
| Digit Span: back+for (total scaled, /19) | 9.9 (2.6) | 10.5 (2.6) | 0.6 (2.2) | 10.4 (2.7) | | 10.0 (3.1) | -0.4 (1.7) | 0.46 (1, 39) | 0.501628613 | **0.38** | | |  |  |  |  |  |  |  |
| Verbal fluency: Letter, number of words a participant named | 10.8 (3.2) | 10.4 (3.1) | -0.4 (3.7) | 10.5 (2.8) | | 11.1 (3.8) | 0.6 (3.6) | 0.38 (1, 39) | 0.541185046 | 0.175 | | |  |  |  |  |  |  |  |
| Verbal fluency: Category, number of words a participant named | 13.4 (5.5) | 13.3 (4.6) | -0.1 (7.5) | 14.5 (4.1) | | 15.2 (5.5) | 0.7 (7.3) | 0.42 (1, 39) | 0.520734516 | **0.375** | | |  |  |  |  |  |  |  |
| Stroop: Accuracy, reaction time | 0.947 (0.046) | 0.971 (0.019) | 0.024 (0.041) | 0.969 (0.041) | | 0.970 (0.035) | 0.002 (0.036) | 0.01 (1, 39) | 0.920856617 | 0.074 | | |  |  |  |  |  |  |  |
| *>0.3, moderate effect | | | | |  | | | |  | |  | | |  |  |  |  |  |  |
